# Genomic Diversity, Competition, and Toxin Production by Group I and II *Clostridium botulinum* Strains Used in Food Challenge Studies

**DOI:** 10.3390/microorganisms10101895

**Published:** 2022-09-23

**Authors:** Brooke Kathryn Bowe, Travis Gwynn Wentz, Brieana Marie Gregg, William Howard Tepp, Kristin Marie Schill, Shashi Sharma, Sabine Pellett

**Affiliations:** 1 Department of Bacteriology, University of Wisconsin-Madison, 1550 Linden Dr, Madison, WI 53706, USA; 2 Food Research Institute, University of Wisconsin-Madison, 1550 Linden Dr, Madison, WI 53706, USA; 3 Microbiology Doctoral Training Program, University of Wisconsin-Madison, 1550 Linden Dr, Madison, WI 53706, USA; 4 Division of Microbiology, Center for Food Safety and Applied Nutrition, Food and Drug Administration, College Park, MD 20740, USA

**Keywords:** *Clostridium*, *Clostridium botulinum*, botulinum neurotoxin, BoNT, whole-genome sequencing, botulism, food challenge, food safety, bacteriocin

## Abstract

Botulinum neurotoxins (BoNTs) produced by the bacteria *Clostridium botulinum* are the causative agent of human and animal botulism, a rare but serious and potentially deadly intoxication. Foodborne botulism is caused by the consumption of foods containing BoNTs, which results from contamination of foods with *C. botulinum* spores and toxin production by the bacteria during growth within the food. Validation of the safety of food products is essential in preventing foodborne botulism, however, limited guidance and standards exist for the selection of strains used in *C. botulinum* food challenge studies. Sequencing and genomics studies have revealed that *C. botulinum* is a large, diverse, and polyphyletic species, with physiologic and growth characteristics studied only in a few representatives. Little is known about potential growth competition or effects on toxin production between *C. botulinum* strains. In this study, we investigated an applied cocktail of ten *C. botulinum* strains, seven Group I and three Group II. Whole genome SNP alignments revealed that this strain cocktail encompasses the major clades of the Group I and II *C. botulinum* species. While growth competition appears to exist between several of the strains, the cocktail as a whole resulted in high levels of BoNT production.

## 1. Introduction

*Clostridium botulinum* is a polyphyletic group of Gram-positive, anaerobic, spore-forming bacteria that is capable of producing botulinum neurotoxins (BoNT), which are the causative agent for the human and vertebrate disease botulism. Botulinum neurotoxins are a large and diverse proteinaceous toxin family and are classified into seven immunologically distinct and highly potent toxin serotypes (A–G), with several serotypes being further subdivided into multiple subtypes denoted by a number after the letter [1]. To date, over 40 BoNT subtypes have been described, which are defined by an amino acid sequence variance >2.6% within the same serotype, and many more variants with lower amino acid variance have been found [1].

BoNTs are the most potent naturally occurring toxins known, with as little as 0.1–1 µg/kg of ingested toxin sufficient to cause human botulism [2]. In the body, BoNTs primarily act at neuromuscular junctions. BoNTs are synthesized as 150 kDa single chain proteins which are endogenously or exogenously cleaved into their active dichain form consisting of a heavy chain (100 kDa) that aids in neuronal cell recognition, receptor binding, and endocytosis, and a catalytic light chain (50 kDa) that, upon entry into the cell cytosol, cleaves SNARE (soluble N-ethylmaleimide-sensitive factor attachment protein receptors) proteins. SNARE proteins are involved in the vesicle docking/fusion complex responsible for neurotransmitter release, and their cleavage by BoNTs disrupts this process leading to descending flaccid paralysis that, in severe cases, may include respiratory failure and death [3,4]. Botulism paralysis is often prolonged for months and may require extensive medical intervention, with pharmaceutical interventions restricted to antitoxin administration that is risky and only effective if given before neuronal cell entry by the toxin [5].

Clinically recognized forms of botulism include infant botulism, foodborne botulism, and wound botulism, with foodborne botulism reported as the second most common cause in the United States and the most common form in most other developed countries [6,7,8]. Foodborne botulism results from the consumption of BoNT that developed within improperly processed or stored food as a result of the germination, growth, and toxin production of contaminating *C. botulinum* spores [9]. Data from the Centers for Disease Control and Prevention (CDC) estimates that foodborne botulism was reported in the United States between 1899 and 1996 with approximately 24 cases per year [6,10] and between 2001 and 2017 with about 19 confirmed cases per year [8]. The low incidence of foodborne botulism today is due to the strict guidelines the food industry must observe to prevent contamination of foods with spores and the potential growth of *C. botulinum* in stored foods.

Similar to the diversity of the BoNTs, the bacteria producing this toxin family also are diverse and span several distinct species amongst the genus *Clostridium* [11]. Historically, *C. botulinum* is separated into four species groups (I–IV) based on physiologic and genetic differences [12]. Current guidelines to prevent foodborne botulism cover both Groups I and II *C. botulinum* strains since strains from both of these groups cause the majority of human botulism cases [13,14]. Strains in Group I (proteolytic *C. botulinum*) strains produce neurotoxin serotypes A, B, or F, which are mesophilic and form highly heat-resistant spores not killed by standard boiling procedures. Strains typically have minimum and optimal growth temperatures of 12 °C and 37 °C, respectively, and can grow at a minimum pH of 4.6. Group II (non-proteolytic *C. botulinum*) strains are psychrotrophs with spores of moderate heat resistance and produce neurotoxin serotypes B, E, or F. These strains generally can grow at a lower temperature range, with minimum and optimal growth temperatures of 3 °C and 30 °C, respectively [3,9].

Geographically, Group I strains are widely distributed in the soils of temperate climates, including the United States, South America, China, and Europe, while Group II strains are generally found in northern aquatic sediments including in Alaska, Canada, Japan, and Northern and Eastern Europe and the contiguous United States [15]. The geographic and physiologic differences between the Group I and Group II strains result in different foods of concern for potential foodborne botulism outbreaks. Group I strain outbreaks are more common in low-acid, home canned foods in the United States, and home cured meats and sausages in Europe [16,17,18]. Group II strains are more of a concern in minimally processed chilled foods or marine products due to their lower threshold temperature for growth [17]. Minimal processing of foods often necessitates testing of the foods with representative strains from both Group I and Group II species.

Production of BoNTs in foods can be prevented by known strategies such as the “botulinum cook”, which is a 3 min heat treatment at 121.1 °C to eliminate proteolytic *C. botulinum* spores or 90 °C for 10 min to eliminate non-proteolytic spores only [19], acidification to a pH below 4.6 [18], a maximum water activity of 0.92 or a salt concentration of at least 10% throughout the food [20], and appropriate storage temperature. However, many food products that may be contaminated with *C. botulinum* spores are not compatible with these methods. Thus, *C. botulinum* food challenge studies remain essential in the food industry to validate the safety of foods, as consumer demands shift towards more minimally processed, ready-to-eat meals.

There are two main types of challenge studies performed to verify that *C. botulinum* toxins will not be present in consumer products, spore inactivation challenge studies and food challenge studies. In inactivation challenge studies, foods spiked with *C. botulinum* spores are processed by methods that potentially inactivate the spores, and surviving cells are counted [16]. There is no acceptable limit for surviving *C. botulinum* spores in inactivation challenge studies, and all spores must be inactivated as an effective prevention strategy [16]. In food challenge studies, foods are inoculated with spores and stored under various storage conditions for prolonged times (1.25–1.5× the shelf life of the food) and assessed for germination, growth, and toxin production in the food product [21]. In food challenge studies, it is the toxin that is detected in foods, not the bacteria, typically using the gold standard toxin detection assay, the mouse bioassay. In vitro assays may be used to screen samples, but presumptive positives must be confirmed using the mouse bioassay [21,22]. It is recommended for food challenge studies to include strains that produce BoNT serotypes A, B, or E, which are most often associated with foodborne botulism cases [10], and to use a “cocktail” of several Group I and/or Group II strains to account for intra-species variability [23,24,25,26]. Thus, the level of confidence in food challenge studies is tied to the level of confidence that the strain cocktail is representative of strains within the diverse *C. botulinum* species that have the capability of contaminating the respective food. An ideal challenge cocktail would include strains that represent the ‘worst case’ scenario for the entire polyphyletic species *C. botulinum*, including strains that have the most resistant spores, germinate most easily, exhibit high toxin production of the most potent toxin subtypes, and have the most robust growth in a variety of matrices and least sensitivities to factors affecting growth or toxin production within each represented clade. In addition, an ideal challenge cocktail for testing in a specific matrix should include type and field strains, in particular, some strains isolated from the same matrix as assessed in the challenge. While a selection of these strains would require extensive studies, our increasing understanding of the genomics and physiology of this polyphyletic species [13,14] now enables renewed evaluations of strains currently used in food challenge studies.

A comprehensive review of prior publications on *C. botulinum* food challenge studies has demonstrated that a large variety of strains are being used with no clear consistency in the strains being chosen [21,26,27,28,29,30,31,32,33,34,35]. Some sets of strains were chosen based on their growth compatibility regarding inter-strain inhibition [33,34] or the strain isolation source [31], while other cocktails were described as having been chosen due to the genetic and physiologic variability between the strains combined with the precedence of being used in challenge studies [27]. If possible, strains previously isolated from the same or a similar food to be tested are included [26]. Lack of antagonism between strains has also been mentioned in the literature or by regulatory agencies as a strain selection criterion [26,33,34,36]. Antimicrobial compounds are a common defense mechanism used by bacteria to promote their success in growth by outcompeting other nearby bacteria, including those from the same species, and come in many different forms such as bacteriocins or enzymes from bacteriophages [37]. These inhibitory compounds are already known to be produced by some strains of *C. botulinum* [38,39,40,41]. Concern that the impact of antimicrobial metabolites or other competitive behavior of selected strains may also affect toxin production through growth competition supports the benefit of investigating antagonistic activity prior to cocktail strain selection. Interestingly, the majority of publications on *C. botulinum* food challenge studies do not provide reasoning behind strain selection and, as such, many regulatory agencies differ in their recommendations [20,25,26,36].

A historic and much-cited review on *C. botulinum* challenge studies recommended that for Group I studies, a mixture containing a minimum of five strains each of type A and proteolytic type B should be used in cocktails, with strains 62A and 213B included in all studies [21]. In the same review, Group II challenge studies were recommended to consist of a minimum of three type E and non-proteolytic type B strains along with one type F strain, with strains Eklund 17B and Beluga E in every study. Indeed, many published challenge tests do already include many of these recommended strains, with 62A commonly being used in Group I studies and strains Eklund 17B and Beluga E in many published Group II tests [27,28,29,30,32,33,34].

In this study, we investigated a 10-strain *C. botulinum* cocktail that is based on these recommendations by Doyle [21] and has been successfully used in food challenge studies for over 20 years. The cocktail is made up of seven Group I strains (56A, 62A, 69A, 90A, 113B, 213B, and Okra B) and three Group II strains (Eklund 17B, Alaska E, and Beluga E) (Table 1). Of these strains, seven have been linked to previous food outbreaks (Table 1). Our data show that even though this strain cocktail was selected based on phenotypic and physiologic characteristics only, it contains representatives of most major clades of Group I and Group II *C. botulinum*. We further show, through the investigation of strain compatibility, that growth competition exists between some of the strains, yet the cocktail as a whole exhibited robust toxin production. Overall, our findings support the inclusion of genomic data combined with toxin production data for strain selection in food challenge studies.

## 2. Materials and Methods

### 2.1. Biosafety and Ethics

Our laboratory and personnel are registered with the Federal Select Agent Program for research involving botulinum neurotoxins (BoNTs) and BoNT-producing species of *Clostridium*. The research program, procedures, documentation, security, and facilities are closely monitored by the University of Wisconsin-Madison and the CDC Select Agent Program. All personnel routinely receive suitability assessments and complete rigorous and continuing biosafety training before participating in laboratory studies involving BoNTs and neurotoxigenic *C. botulinum*. All animal studies conducted were approved by the University of Wisconsin-Madison IACUC.

### 2.2. Bacterial Strains and Growth Conditions

The Group I *C. botulinum* strains 56A, 62A, 69A, 90A, 113B, 213B, and Okra B and Group II *C. botulinum* strains Eklund 17B, Alaska E, and Beluga E used in this study came from the Pellett lab culture collection. All strain manipulations were conducted in an anaerobic chamber (Thermo Forma Anaerobic System model 1025) unless specified otherwise. All bacterial media components and chemicals were purchased through Sigma (St. Louis, MI, USA) or Difco (BD Becton Dickinson and Company, Spark, MD, USA). Liquid cultures were grown in TPGY (5% Trypticase peptone, 0.5% Bacto peptone, 0.4% glucose, 2% yeast extract, 0.1% L-cysteine HCl [pH 7.3–7.4]) medium, Toxin Production Medium (TPM; 2% Trypticase peptone, 0.5% glucose, 1% yeast extract [pH 7.3]), or Reinforced Clostridial Medium (RCM). Following preliminary tests, strain Alaska E was found to grow differently in TPGY than the other nine strains, often expressing a non-motile phenotype comprised of long, non-sporulating rods, and was unable to grow a lawn on plates when the liquid culture was grown in TPGY medium. As a result, strain Alaska E was grown in RCM and for a shorter duration for inhibition testing. Liquid cultures of Group I strains were incubated at 37 °C, while liquid cultures of Group II strains were incubated at 30 °C, unless otherwise specified. Strains were grown on 1.5% TPGY agar plates or 1.5% RCM agar plates with a triple antibiotic cocktail of 1.5 µg/mL trimethoprim, 250 µg/mL cycloserine, and 20 µg/mL sulfamethoxazole to select single colonies for sequencing. Plates were streaked in a biologic safety cabinet followed by anaerobic incubation in the anaerobic chamber.

### 2.3. Whole Genome Sequencing

The 10 strains sequenced in this study were grown overnight at 30 °C (Group II strains) or 37 °C (Group I strains) prior to lysis. Genomic DNA was extracted as described in the Gram-Positive protocol for the DNeasy Blood and Tissue Kit Handbook (Qiagen, Hilden, Germany). Genomic DNA was prepared for sequencing using the Illumina DNA Prep kit, Nextera DNA CD Indexes, and sequenced on an iSeq100 sequencer (iseq i1 Reagent v2 (300-cycle)) under manufacturer specifications (Illumina, San Diego, CA, USA).

### 2.4. Core Genome SNP Analysis and Phylogeny

The program panX (v1.6.0, default settings) was used to assign genes from Group I *C. botulinum* and *Clostridium sporogenes* (*C. sporogenes*) (GI/sporo) and Group II *C. botulinum* (GII) into core and accessory genomes [49]. The resulting core genome analysis was used to construct SNP (single nucleotide polymorphism) phylogenies from non-duplicated core genes present in all species present in each species group (GI/sporo: 726, GII: 1778). The GI/sporo strains include 241 Group I *C. botulinum* and *C. sporogenes* that were identified via an MLST scheme described previously [50,51], the 7 Group I strains sequenced as part of this project, and strains NCIB 4301 (GCA_011017885.1), 53B (GCA_011014035.1), and 69A (GCA_000439655.1) which were included due to prior use in food challenge studies [30,32]. The only previously available assembly of strain 69A, GCA_000439655.1, was included in the core genome SNP (cgSNP) analysis due to study relevance despite possessing an elevated pseudogene count. Strains present in the Group II cgSNP analysis included all closed genomes on NCBI/Genbank (4/2021) and additional strains were selected based on additional criteria, including use in food challenge studies and strain diversity based on prior phylogenetic studies [13,27,28,29,31,32,33,34]. Toxin gene presence/absence was predicted via tBLASTn [52]. The panX sequence alignment for the lysin CBO1751 (Genbank accession CAL83288.1) was used to identify its presence across the strains included in the GI/sporo phylogeny. The protein alignment of lysin CBO1751 was extracted from the GI/sporo accessory genome. All phylogenetic trees were visualized via iTOL [53]. All strains and accession numbers are provided in Supplementary Table S1.

### 2.5. Strain Inhibition Testing

To test for antagonism between strains in the cocktail, strains were grown in TPGY medium for 24 h with the exception of strain Alaska E, which was grown in RCM for 16 h to support better growth. Initial inhibition testing was performed as a well diffusion assay. Each culture (0.5 mL) was spread on top of TPGY plates with 1.5% agar as an indicator strain, and the plates were left right side up for 30 min until dry. A total of ten wells were punched into each plate and each of the 10 strains was added to one well per plate at a volume of 40 µL. Once the culture had soaked into the agar (~30 min), plates were incubated upside-down anaerobically at 30 °C. Plates were imaged on an Azure c600 imaging system using Azure spot software version 2.0.062 (Azure Biosystems, Dublin, CA, USA) and analyzed using ImageJ by measuring the area of inhibition. Each indicator strain was tested in triplicate, and the average radius of inhibition between the edge of the well and the zone of lawn clearing was determined.

For additional inhibition testing, each strain was streaked out with a 10 µL inoculation loop on its own plate in a long transverse line as an indicator strain, with all other strains then struck out in perpendicular lines to the middle indicator culture using a 1 µL inoculation loop for each streak. Plates were incubated anaerobically at 30 °C in an anaerobic chamber and imaged on an Azure c600 imaging system daily for up to five days following plating. In a variation of this assay, the TPGY plates were prepared at pH 6.0, and strain Alaska E was used as the indicator strain.

### 2.6. Toxin Production Testing

Strains 56A, 62A, 69A, 90A, 113B, 213B, Okra B, Eklund 17B, Alaska E, and Beluga E were grown in TPGY tubes at 30 °C for 16 h. TPM (10 mL) tubes were inoculated with the TPGY cultures at a volume corresponding to 0.01 OD600 (optical density at 600 nm) per strain, respectively, in the following combinations: 1. each strain by itself, 2. the entire 10-strain cocktail, 3. the 10-strain cocktail missing strain Alaska E (9-strain cocktail), 4. all seven Group I strains, 5. all three Group II strains. All TPM tubes were incubated for 4 days at 30 °C.

To examine toxicity produced by the strain cocktails, a mouse bioassay was performed on the following samples as previously described [54,55]: the 10-strain cocktail (−/+ trypsin), 9-strain cocktail lacking strain Alaska E (−/+ trypsin), 7-strain Group I cocktail (− trypsin), 3-strain Group II cocktail (−/+ trypsin), and the high toxin-producing strain 62A (− trypsin) as a control. Briefly, each sample was incubated for one hour in a 30 mM sodium phosphate buffer (pH 6.3) with 0.2% gelatin (GelPhos buffer), either with no trypsin added or, as indicated, with the addition of trypsin solution (0.5 g Difco 1:250 trypsin per 10 mL H_2_O) as described in the BAM recommendations [16]. Each sample was centrifuged to remove cell pellets, and three-fold serial dilutions of sample supernatants were prepared using GelPhos buffer before injecting eight groups of three female ICR mice (Envigo, Indianapolis, IN, USA, 18–22 g) intraperitoneally with 0.5 mL/mouse with the dilutions, respectively. Mice were observed for 4 days, and the mouse lethal dose of 50 (mLD_50_/mL), at which 50% of mice died, was determined using GraphPad Prism 6 software (nonlinear regression analysis, variable slope, four parameters).

To observe which BoNT serotypes were produced by the cocktails and examine toxin production levels of each strain, a Western blot analysis was performed. Whole cultures of each cocktail and individual strains were grown for 4 days in TPM and were mixed with 4× Laemmli sample buffer to 1× and heated at 70 °C for 10 min, with the addition of 100 mM DTT to create reduced samples where indicated. Western blots were probed using 0.25 µg/mL polyclonal affinity-purified rabbit IgG specific to BoNT/A1, BoNT/B1, or BoNT/E3 that were prepared in our laboratory [56], and a 1:50,000 dilution of HRP conjugated goat-anti-rabbit secondary antibody (Azure Biosystems, Dublin, CA, USA). Western blots were imaged using an Azure c600 imaging system and with a chemiluminescent SuperSignal West Dura substrate (Thermo Fisher Scientific, Waltham, MA, USA). All individual strains were tested in two independent experiments, and the cocktails were all tested in triplicate. Bands on the Western blot of the triplicate cocktail samples were quantified by densitometry using AzureSpot, and graphs showing the average and standard deviations were prepared in Excel. Statistical significance was determined by a pairwise two-tailed t-test, and results were considered significantly different at a *p*-value < 0.05.

### 2.7. Specific Toxin Activity Determination of BoNT/B4 and BoNT/E3

Purified 150 kDa neurotoxins from *C. botulinum* Alaska E, Beluga E, and Eklund 17B were produced by previously described methods [57] with minor modifications for strains Beluga E and Eklund 17B. Briefly, the pH of a 72 h Beluga E culture was lowered to pH 3.8 before centrifugation, whereas the 72 h culture of Alaska E was centrifuged without lowering the pH. Purification of BoNT/B4 from strain Eklund 17B followed the method of Alaska E with a change in pH from 7.4 to 8.0 for the DEAE (diethylaminoethyl-cellulose) chromatography complex breaking step and the addition of a pABTG (aminophenyl-ß-D-thiogalactopyranoside) chromatography step immediately following the DEAE pH 8.0 chromatography. The Beluga E and Alaska E complex (toxin + NTNH [nontoxic-nonhemagglutinin]) in 25 mM sodium citrate (pH 6.0) were activated by treatment with trypsin at a complex to trypsin ratio of 30:1 for 30 min at 37 °C prior to the final isolation of 150 kDa toxin. Purified BoNT/B4 from Eklund 17B was trypsinized in 50 mM sodium phosphate buffer (pH 6.0) at a toxin to trypsin ratio of 25:1 for 30 min at 37 °C. In both cases, the trypsin digestion was stopped by the addition of a soybean trypsin inhibitor (SBTI) at a ratio of 4:1 SBTI to trypsin. Purified toxins were analyzed by SDS-PAGE for purity, and the specific activity of the purified BoNT/E1,/E3, and/B4 was determined by mouse bioassay as previously described [54]. Serial dilutions of the respective toxins were prepared in 30 mM GelPhos buffer (pH 6.3). Six groups of four mice were injected intraperitoneally with 0.5 mL/mouse with the toxin dilutions, respectively, and mice were observed for 4 days. The activity expressed as pg toxin/LD_50_ was determined based on the method of Reed and Muench [58].

## 3. Results

### 3.1. Phylogenetic Analysis of Cocktail Strains Indicates Some Lineages of Toxigenic Clostridium Are Not Represented

**Figure 1:**
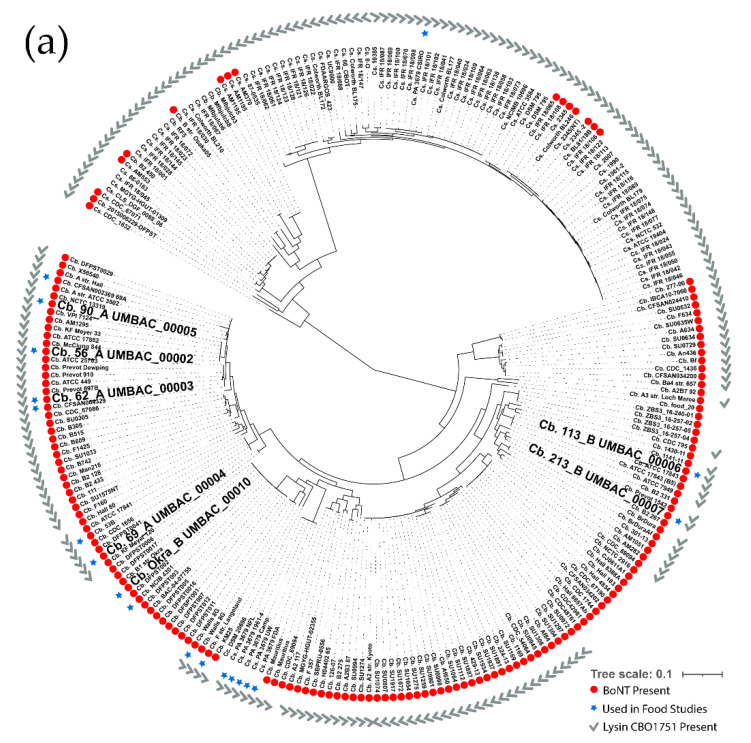

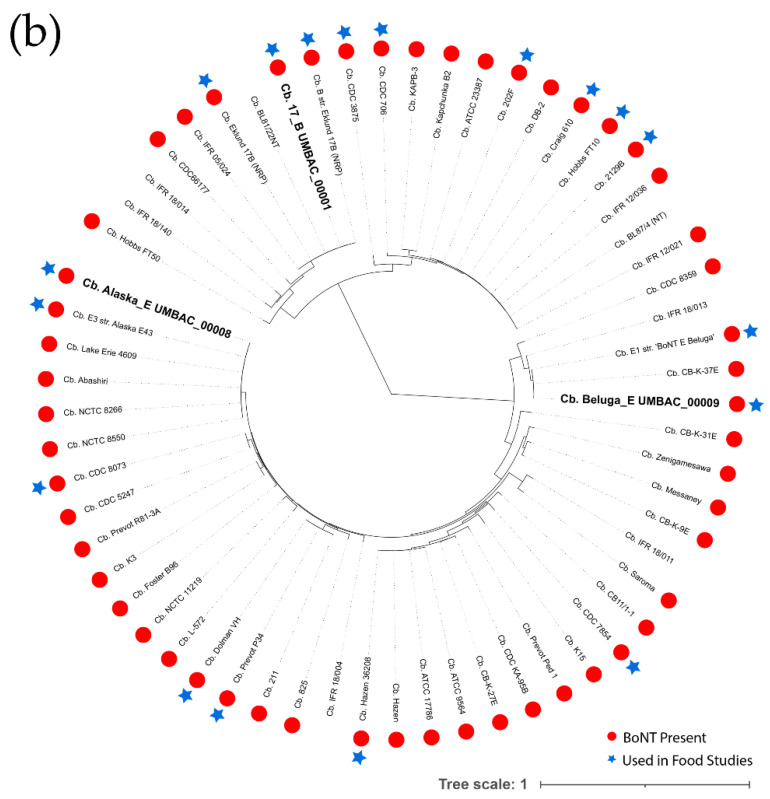
*C. botulinum* Group I/*C. sporogenes* and *C. botulinum* Group II phylogenies. Core genome phylogenies demonstrating the relationship of sequenced cocktail strains (bold) relative to other strains of (**a**) Group I *C. botulinum* and *C. sporogenes*, and (**b**) Group II *C. botulinum*. The red dots indicate the presence of toxin gene clusters and the blue stars indicate strains commonly used in food safety studies. The grey checkmark in (**a**) denotes the presence of a CBO1751 gene homolog.

The core genome phylogeny of *C. botulinum* Group I and the closely related *C. sporogenes* species revealed that the 7 Group I strains of the 10-strain cocktail are distributed throughout the major clades, with the exception of one major unrepresented clade and several sub-clades (Figure 1a). Group I strains used by other groups in food safety studies [30,32] were closely related to the strains used in the 10-strain cocktail. In addition, several isolates of *C. sporogenes* PA3679 (FDA, UW, Camp, 1961-4, and NFL) grouped into the *C. botulinum* species, whereas another isolate of PA3679 grouped into *C. sporogenes*, consistent with previous in-depth analyses of this strain [59,60]. Multiple sequence alignment of the gene CBO1751 [61], as part of the pangenomic analysis, revealed that 214 of the 251 Group I and *C. sporogenes* strains contain one or more homologs of this gene, including all of the Group I strains contained within the 10-strain cocktail studied here, with the exception of strain Okra B (Figure 1a). The Group II phylogeny revealed that the 3 Group II strains used in the 10-strain cocktail represent three of the four major clades of the Group II phylogeny, yet several sub-clades are not represented (Figure 1b). Group II strains reported in other food safety studies [27,28,29,31,32,33,34] span the entire Group II phylogeny, although it should be noted that the strains are derived from multiple studies, none of which, to the best of our knowledge, used a cocktail encompassing strains from each major clade.

### 3.2. Several Group I and Group II C. botulinum Strains Display Antagonistic Growth Behavior

Since strains within a cocktail may compete against each other, as indicated by the presence of a predicted lysin gene CBO1751 homolog in most Group I strains (Figure 1a) along with a recent report that strain Alaska E can produce a bactericidal lysin [39], we investigated potential inhibitory behavior between the strains of the 10-strain cocktail in a well diffusion assay. After 2 days, several clear zones indicative of inhibition between the test strains in the wells and the indicator lawn were observed (Table 2, Figure S1). All of the Group I strains except Okra B had clear zones of varying sizes surrounding their wells, indicating inhibition against at least one other strain in the cocktail. While the smaller clear zones of less than 2 mm may indicate mild growth competition between the strains, larger clear zones likely indicate the production of a lysin, phage, bacteriocin, or bacteriocin-like compound. Strains 213B and 113B exhibited the largest clear zones, whereby strain 213B was inhibitory against all other Group I strains and 113B clearly inhibited strains 62A and 90A. This is consistent with a previous report that identified a bacteriocin gene (boticin B) in strain 213B [38] as well as the finding that strains 213B and 113B each contain a homolog to the known lysin CBO1751 [61] with ~86% amino acid identity and 100% coverage, respectively (Figure 1). Additionally, strain 213B was capable of mildly inhibiting one Group II strain, Alaska E (Table 2, Figure S1). Strain 90A also showed clear zones against all other Group I strains, although most were mild (<2 mm). The proteolytic type B strains and strain 69A were each inhibited by most, if not all, of the type A strains in the cocktail and by strain 213B. None of the Group II strains showed any clear zones against any of the Group I or Group II strains under the tested conditions. The well diffusion assay results were confirmed by a culture-streak assay which demonstrated growth competition between the same strains (Figure S2).

**Figure 2:**
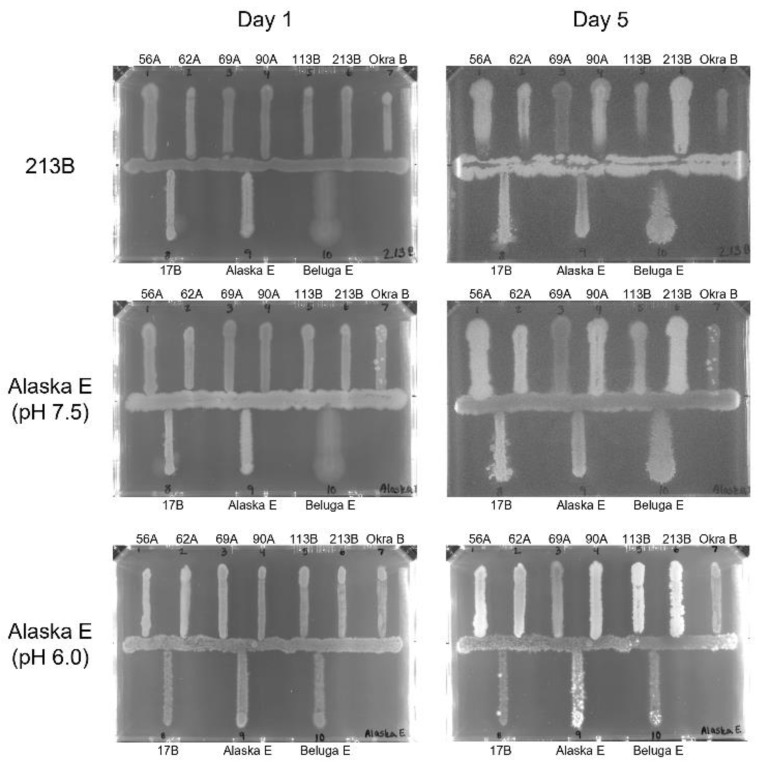
Growth inhibition patterns of *C. botulinum* strains. Representative inhibition plates with center indicator strain 213B on plates with pH 7.5 and Alaska E on plates with pH 7.5 and 6.0 on day 1 and day 5 of incubation.

These data largely agree with the literature, which has described bacteriocin activity associated with strain 213B [38], and with our findings that putative homologs to the CBO1751 gene are present in all Group I strains except Okra B. However, a recent publication has also identified an inhibitory endolysin (LysB) in strain Alaska E with activity against clostridia and other gram-positive bacteria [39], yet we observed no inhibitory activity by strain Alaska E despite confirming through genomic sequence analysis that the isolate we used does contain the LysB gene. Since this study by Morzywolek et al. suggested peak inhibitory activity by LysB at pH 6.0–6.5, and our plate assays were conducted on agar plates of pH 7.3–7.4, an additional streak assay was conducted with strain Alaska E as the centered indicator strain on TPGY plates at pH 6.0. After up to 5 days of incubation, only mild inhibition was observed against strain 113B (Figure 2).

### 3.3. A 10-Strain Cocktail Combining Group I and Group II Strains Produces High Levels of Toxin in Spite of Competition

Since food challenge assays use the detection of toxin as an endpoint, competition of strains within a cocktail may not be detrimental as long as some of the strains grow and produce sufficient amounts of toxin. In order to examine toxin production of the strain cocktails versus individual strains, a mouse bioassay, and Western blots were conducted on individual strains and cocktails grown for 4 days in TPM at 30 °C, respectively. The toxin activity was determined by mouse bioassay for the untrypsinized and trypsinized 10-strain cocktail, 10-strain cocktail without strain Alaska E, Group I cocktail, and Group II cocktail. Strain 62A was used as a control strain for high toxin production [62]. The specific activity of the seven-strain Group I cocktail and strain 62A were similar, with both determined to be 1.1 × 10^6^ mouse LD_50_/mL (mLD_50_/mL) (Table 3), indicating no effect on overall toxin activity by combining these seven Group I strains, in spite of the observed inhibitory activity between the strains (Table 2). While the untrypsinized 10-strain and 9-strain cocktails had an ~10-fold lower specific activity of 4.3 × 10^4^ mLD_50_/mL each, trypsinization increased the activity of the 10-strain cocktail to 3.8 × 10^5^ mLD_50_/mL and of the 9-strain cocktail to 1.1 × 10^6^ mLD_50_/mL (Table 3). This suggests that when grown at 30 °C, the majority of the toxin activity in the 10-strain cocktail is derived from Group II strains, which require trypsinization prior to the mouse bioassay for full activity, and that the presence or absence of strain Alaska E has only a minor effect on overall toxin production. The Group II cocktail also showed robust toxin activity of 1.2 ×10^5^ mLD_50_/mL, which required trypsinization, as expected.

**Figure 3:**
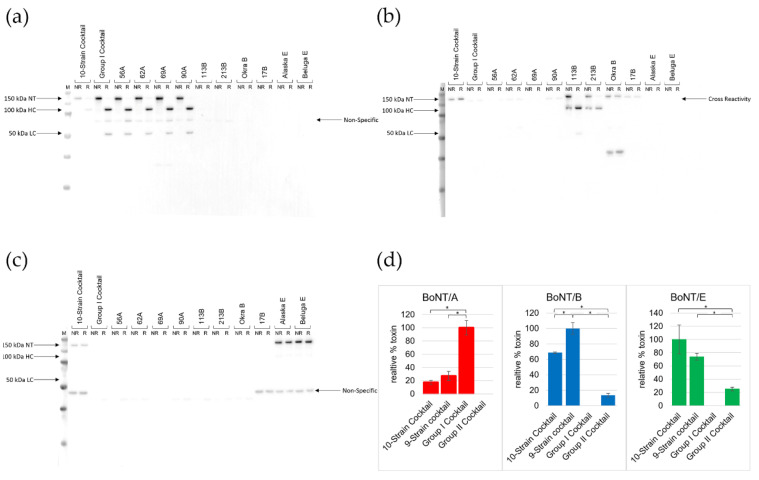
Toxin production by *C. botulinum* strain cocktails and individual strains. (**a**–**c**) Western blot analysis of non-reduced (NR) and reduced (R) whole culture samples alongside a molecular weight marker (M; PageRuler Plus) grown for 4 days at 30 °C in TPM. The toxin type produced was detected with (**a**) BoNT/A1-specific antibody, (**b**) BoNT/B1-specific antibody, and (**c**) BoNT/E3-specific antibody. Non-specific bands can be faintly seen in each blot and, in the blots probed with antibodies specific to BoNT/B1, there appears to be cross-reactivity to the 150 kDa type A neurotoxin rather than the BoNT/B. It is this cross-reactivity that can be seen in the Group I cocktail samples on the blots probed with BoNT/B1-specific antibody. (**d**) Quantitative depiction of the relative percentage of each toxin type produced by each cocktail (n = 3). The cocktail that produced the highest amount of the given BoNT serotype was set at 100%. Asterisk (*) indicates a statistical significance with *p* < 0.05.

Toxin production was further investigated by Western blots, which showed strong toxin production for each individual strain, but predominantly type A toxin in the Group I cocktail and type B and E toxin in the 10-strain cocktail (Figure 3a–c). Interestingly, examination of non-reduced versus reduced samples revealed full proteolytic processing to the dichain form of the toxin for all type A toxins, but no processing for the type B and E toxins detected in the 10-strain cocktail (Figure 3b,c), which is consistent with the increase in toxin activity after trypsin treatment (Table 3). While strains 113B and 213B showed complete processing of their type B2 toxin, the BoNT/B1 produced by the third proteolytic type B strain, Okra B, did not reduce to the 100 kDa heavy chain (HC) and 50 kDa light chain (LC), indicating no toxin processing by strain Okra B. It is thus unclear whether the non-processed type B toxin detected in the 10-strain cocktail is predominantly derived from the Group I strain Okra B or Group II strain Eklund 17B, or from both. Since Eklund 17B produces a subtype B4 toxin and the polyclonal antibody used in this study was raised against BoNT/B1, antibody recognition of the two subtypes was directly compared by Western blot, showing that BoNT/B4 is recognized about half as efficiently as BoNT/B1 by Western blot (data not shown). A previous study has indicated that BoNT/B2 is recognized robustly by polyclonal antiserum raised against BoNT/B1, although some immunological distinctions were demonstrated using monoclonal antibodies [63]. A similar analysis of the antibody raised against BoNT/E3 showed no difference in the detection of BoNT/E1 and BoNT/E3 (data not shown).

Toxin type expression in each of the examined strain cocktails was further investigated quantitatively with triplicate samples grown at 30 °C for 4 days (Figure S3). Comparing toxin type levels between the various cocktails confirmed that the BoNT/A production in the 10- and 9-strain cocktails were reduced 4–5 fold compared to production in the Group I cocktail. Meanwhile, BoNT/B production was not detectable in the Group I cocktail but was relatively strong in the 10- and 9-strain cocktails (Figure 3d), yet decreased compared to production by the strongest producing individual strain 113B (Figure 3b). Interestingly, BoNT/E production was increased 3–4 fold in the 10- and 9-strain cocktails compared to the Group II cocktail (Figure 3d), while toxin production in individual strains was significantly stronger than in any cocktail (Figure 3c). These data indicate significant changes in toxin production by individual strains within the 10-strain cocktail, yet the mouse bioassay data showed the maintenance of strong overall toxin activity.

Monitoring of the OD600 during the first 3.5 h and a final pH and OD600 reading taken at 4 days of growth showed that strains 56A, Eklund 17B, and Okra B began growing at the fastest rate between hours 0 and 3.5, whereas strains 90A, 213B, Alaska E, and Beluga E had very slow initial growth rates (Figure S4a). The 10- and 9-strain cocktails exhibited a fast initial growth rate, which was only slightly reduced compared to the added rates of all individual strains within the cocktail, whereas the Group I cocktails had a growth rate of ~0.06 OD600/h, which is about half of the sum of the initial growth rates of the individual strains within that cocktail (~0.13 OD600/h), suggesting minor competition of the strains (Figure S4a). The Group II cocktails had the slowest initial growth, yet it was similar to that of the sum of the individual non-proteolytic strains, suggesting no competition within the Group II strain cocktails. The final OD600 after 4 days of growth was around 1 for all individual Group I strains and the Group I cocktail, reflective of cell lysis that has occurred. For all Group II strains as well as all cocktails containing Group II strains, the OD600 was above 2.5, reflective of the lack of cell lysis of non-proteolytic strains and indicative of strong growth of the non-proteolytic strains in the 10- and 9-strain cocktails (Figure S4b). The final pH of the Group I strain cultures was between 6.20–6.34 and between 5.08–5.12 for the Group II strain cultures. The 10- and 9-strain cocktails had a pH in between these two ranges, with an average pH of 5.87 for the 10-strain cocktails and an average pH of 5.91 for the 9-strain cocktails, indicating growth of both Group I and Group II strains (Table S2).

### 3.4. BoNT/E1 and/E3 Have Similar Specific Activities, While BoNT/B4 Has Reduced Activity Compared to BoNT/B1

Analysis of the sequences of strains used in this study indicated that most strains harbored the A1, B1, or E1 toxin types, but strain Beluga E produces subtype E3, strain Eklund 17B produces subtype B4, and strains 213B and 113B produce the B2 toxin. Previous studies have shown that subtypes of BoNTs can vary in toxin potency, and, in fact, BoNT/B2 was found to have about a 10-fold lower potency than BoNT/B1 [63]. Since the most frequently used endpoint assay in food challenge studies is the mouse bioassay, we were interested in investigating the specific activity of BoNT/E3 and B4 compared to their subtype 1 prototypes. Mouse bioassays of purified 150 kDa toxins determined a specific activity of 13 pg/LD_50_ for BoNT/E3 and 16 pg/LD_50_ for BoNT/E1, which is similar to previously reported specific activities for these two toxin subtypes [64]. In addition, the activity in cultured neurons as well as the duration of action was similar for these two BoNT/E subtypes (data not shown), indicating similar toxicity for both subtypes. For BoNT/B4, however, the specific activity of purified 150 kDa toxin was determined to be 1 ng/LD_50_ non-trypsinized, and after trypsin, treatment activity increased to 100 pg/LD_50_. This is approximately 10- to 20-fold lower than that typically reported for BoNT/B1 [64].

## 4. Discussion

Whole genome sequencing of an increasing number of *C. botulinum* strains is changing the landscape of the *C. botulinum* and BoNT research field. The sequences for over 1000 strains of *C. botulinum* are currently available, and bioinformatics analyses are revealing previously uncovered insights into aspects of strain relatedness, lateral gene transfer, immune defenses, evolution, and physiology of the strains [13,14,39,51,59,61,65]. These data are of particular interest for food safety studies, which for decades have relied on assays utilizing select strains to represent the entire species. In this study, we performed genomic analyses combined with growth and toxin production studies to investigate strain interactions and toxin production within a 10-strain *C. botulinum* cocktail frequently used in food challenge studies. This strain cocktail was originally selected based on the limited information available at the time, including strain isolation source and toxin serotype production [21], but with no knowledge of the genomic phylogeny of *C. botulinum*. Numerous phylogenetic studies have since been performed observing the relationship between toxin type and strain source in foodborne botulism cases. In Group I, isolates from foodborne botulism appear to produce a wide variety of toxin subtypes, although BoNT/A1, A2, B1, B2, and B5/F2 have each been recorded in multiple cases of foodborne botulism [14]. Group II foodborne botulism isolates have most frequently been found to produce subtypes BoNT/B4, E1, and E3 [13]. Occasionally, other toxin subtypes have been identified from both Group I and II, and some isolates from food have contained two toxin types [13,14,66]. Recently, extensive sequence and bioinformatics analyses of *C. botulinum* strains have revealed the intraspecies diversity of both Group I and Group II *C. botulinum* and the BoNTs they produce [3,13,14]. These analyses also revealed the presence of strains detected in foodborne botulism outbreaks in most clades of both Group I and Group II strains but suggested the possibility that certain phylogenetic lineages within the species may have a higher association with foodborne botulism outbreaks than others [13,14].

With these data in mind, it has previously been proposed to consider not only physiologic differences but also the genomic distribution of strains for food safety studies [36,67]. Genomic SNP phylogenetic analysis of the 10-strain cocktail used in this study showed that the strains were predominantly distributed into the same lineages that Brunt et al. determined to have higher numbers of food isolates (Figure 1) [13,14]. One exception was strain Eklund 17B, which fell into a clade not containing strains previously associated with foodborne botulism [13], although this could simply be due to such strains having not yet been identified. While, overall, the 10-strain cocktail in this study appears to encompass most clades within the Group I and Group II *C. botulinum* species, at least one major sub-clade in Group I and Group II, respectively, were not represented (Figure 1). Previous studies have found strains associated with foodborne botulism within these clades [13,14], but it is not known whether the strains within those clades have distinct physiologic characteristics. Interestingly, additional strains not contained in this study but commonly used in food challenge assays fall into the same clades as the strain cocktail examined here for Group I, whereas for Group II, additional strains are widely distributed throughout all major clades (Figure 1) [27,28,29,30,31,32,33,34,35].

The Group I phylogeny additionally emphasizes that Group I *C. botulinum* and *C. sporogenes* are distinct species, raising concern for the idea of using *C. sporogenes* as an atoxic surrogate in food studies. The commonly used “*C. sporogenes*” strain PA3679 (isolates FDA, UW, Camp, 1961-4, and NFL), however, are genetically Group I *C. botulinum* strains as previously described [59,60], and potentially an appropriate atoxic Group I surrogate. Strain PA3679 has previously been found to have high spore heat resistance characteristics, ideal for spore inactivation studies. However, it was also found that several strains exist with the same name (isolates 1961-2, 1990, and 2007) which exhibit significantly lower spore resistance and were genetically more similar to *C. sporogenes* strains than the isolates FDA, UW, Camp, 1961-4, and NFL [59], which all grouped within the *C. botulinum* clade (Figure 1a). Similarly, in our phylogeny, isolate PA3679 CSIRO is grouped into the distinct *C. sporogenes* clade (Figure 1a). These data underscore the importance of considering genomics when selecting *C. botulinum* or surrogate strains in food safety studies.

While the diversity of strains selected for food challenge cocktails is an important consideration, previous studies have suggested that it is also important to ensure that inhibition between strains is minimized, as the antagonistic activity may alter toxin production [67,68]. Two strains within the strain cocktail studied here have previously been reported to contain genes that encode inhibitory compounds, the boticin B from strain 213B [38] and the endolysin LysB from strain Alaska E [39]. In addition, a recent study has identified a lysin, CBO1751, in *C. botulinum* strain ATCC3502 with activity against Group I *C. botulinum* [61,69]. A panX genome analysis indicated that a potential homolog to CBO1751 exists in 214 of the 251 Group I strains analyzed in this study, including all Group I strains contained within the 10-strain cocktail except Okra B (Figure 1a). Direct testing of the antagonistic growth behavior of the strains in the 10-strain cocktail by a well diffusion assay and by streaked plates revealed that considerable antagonistic growth behavior occurs between the Group I strains, with strains 213B and 113B exhibiting the strongest apparent inhibitory activity (Table 2). Strain Alaska E, however, did not inhibit any of the other strains in either the well diffusion or supplementary streaked plate testing, with only slight growth competition between strains Alaska E and 113B appearing even when the pH of the plate was lowered to pH 6.0 to allow for optimal endolysin activity (Table 2, Figure 2) [39]. This lack of inhibition occurred despite confirming that the Alaska E isolate used in our study did contain the LysB gene via tBLASTn with 100% shared identity. The lack of inhibition by strain Alaska E in our study could be due to low production or poor secretion of the endolysin, as in the previous study the endolysin was tested after recombinant expression rather than being produced by the strain directly [39]. Another possibility is that endolysin is more effective at preventing spore germination [39], which was not examined in this study. Overall, the inhibition data are consistent with a previous study by Eklund et al. observing similar patterns of inhibition between Group I and Group II *C. botulinum* strains [68].

Despite apparent growth competition or inhibition between the strains, examination of toxicity produced by the 10-strain cocktail revealed there was only a minor decrease in the total toxicity compared to the individual strong toxin-producing strain 62A (Table 3). This suggests that despite some antagonistic behavior between the strains, the toxin production remains strong for the overall cocktail. Interestingly, though, both the requirement of trypsin treatment for full toxin activity and the toxin analyses by Western blot suggests that the majority of toxin activity in the 10-strain cocktail is derived from the non-proteolytic cultures. Furthermore, it appears that the toxin production of the type B strains was inhibited by the type A strains under the tested conditions, as no BoNT/B was detected by Western blot in the Group I cocktail (Figure 3b), aligning with the results of the inhibition assay showing mild antagonistic behavior of several type A strains against the proteolytic type B strains (Table 2). In contrast, in the 10-strain cocktail, BoNT/A production was decreased compared to the Group I cocktail, and instead more BoNT/B and BoNT/E were produced (Figure 3), suggesting stronger toxin production by Group II strains within a combined cocktail grown for 4 days at 30 °C in TPM. Since the reduction in the type B toxin detected in the 10-strain cocktail did not result in separate 100 kDa and 50 kDa heavy and light chain bands as is typical for a proteolytically processed toxin from Group I strains (Figure 3b), it is likely derived from the non-proteolytic strain Eklund 17B. However, since proteolytic strain Okra B also does not appear to proteolytically process its type B toxin, it cannot be ruled out that Okra B is the source of the type B toxin in the 10-strain cocktail. Overall, our toxicity data suggest that, under the tested conditions, in strain cocktails combining Group I and Group II *C. botulinum*, toxin activity is primarily due to toxins produced by the Group II strains, whereas in Group I strain cocktails, type A toxicity is predominantly produced. This is similar to what Eklund et al. observed in that the type A strains outcompeted the proteolytic type B strains for toxin production when proteolytic strain 62A was co-incubated with proteolytic strain 9B or 53B [68]. In addition, when proteolytic strain 62A was co-incubated with non-proteolytic strain EF4 at 30 °C, greater amounts of type E toxicity were observed after 3 days of growth, although when the co-culture was grown for another 1–2 weeks, the type A toxin titer increased, whereas the type E toxin titer decreased, which the authors attributed to the presence of the proteolytic enzymes in the cocktail [68].

Monitoring of the early growth of the strains during the toxin production study showed significant differences in growth rate, with the type E strains growing most slowly, yet they did ultimately grow to high density with strong toxin production both alone and in the 10-strain cocktail (Figure 3 and Figure S4). This could be due to a variety of reasons, such as varying growth competition in the presence of multiple strains, physiological differences that can affect toxin production or stability based on the environmental conditions, or the creation of an environment by the Group I strains that favors Group II growth, toxin production, or toxin stability when all 10 strains are grown together.

Previous studies have shown that toxin potency differences can exist between different BoNT subtypes within one serotype [63,70,71,72,73,74]. For example, BoNT/A1 has about 1000-fold higher activity in mice compared to BoNT/A4, while BoNT/A3 appears to have a shorter duration of action than other BoNT/A subtypes 1–5 [70,71]. While genomic analyses of the 10 strains investigated here showed that most strains contained the ‘prototype’ subtype of their respective toxin serotype, there is the exception of Alaska E, which produces the E3 subtype, and Eklund 17B, which produces the B4 subtype. A review article summarizing previously reported BoNT subtype activities noted a three-fold lower potency of E3 compared to E1 [64], however, we determined the same specific activity for isolated 150 kDa BoNT/E3 from strain Alaska E and BoNT/E1 from strain Beluga E, suggesting no issues when using either of these strains with regards to toxin activity detection. BoNT/B4, however, had not previously been characterized, and our finding that the specific activity of isolated and trypsinized BoNT/B4 was about 10–20-fold lower than previously described for BoNT/B1 should be considered when toxin activity is used as an endpoint for food challenge studies with strain cocktails including this strain. The reporting of foodborne botulism cases caused by strains expressing BoNT/B4 [13] supports its inclusion.

Taken together, our data suggest that while some growth competition exists between the strains contained within the examined 10-strain cocktail, overall toxin production levels remain high. Foods in which challenge studies detect any type or quantity of BoNT are considered unsafe under the tested conditions. Thus, while the dominant toxin type produced may change over time and in different matrices, the overall toxicity of this 10-strain cocktail supports its use in food challenge studies. However, our studies suggest the following considerations: 1. When testing foods primarily at risk by Group I *C. botulinum*, the Group I only cocktail may be advantageous to allow for optimal growth of members of this species in the respective food matrix without potential interference by Group II organisms; 2. The presence or absence of a bacteriocin or lysin gene should not preclude a strain from being used in a challenge cocktail, as a large number of strains contain potential lysin genes and their effect on toxin production is not consistent with inhibitory activity; 3. Toxin detection assays that utilize antibodies for detection, such as ELISA, must always examine the production of all toxin types potentially produced by the strain cocktail, as competitive or antagonistic behavior of the strains within the cocktail under the specific testing conditions may result in the production of primarily one toxin type; 4. Toxin subtypes should be considered when selecting strains, as the currently used toxin activity assay (mouse bioassay) is dependent on toxin potency which can vary for toxin subtype; and 5. The phylogenetic studies indicate that the addition of or replacement of individual strains within the cocktail to encompass all phylogenetic clades may be considered, although physiologic characteristics, if available, and prior association with foodborne botulism, should remain the predominant factor in strain selection. As more and more in vitro methods are being developed for BoNT detection in an effort to reduce or replace the mouse bioassay, including ELISA, endopep-MS, and others, considerations of strain selection and consequential type-specific toxin detection remain important aspects. Currently, the FDA maintains the mouse bioassay as the gold standard assay for botulinum neurotoxin detection in food samples, and although in vitro assays may be utilized, any positive samples must be confirmed with the mouse bioassay.

In summary, our findings suggest that mild competitive or inhibitory activity between *C. botulinum* Group I strains may be widespread, as most strains contain putative homologs to the known lysin CBO1751 and antagonistic activity of various degrees was observed between most *C. botulinum* Group I strains examined in this study. No growth inhibitory activity was observed between individual Group II strains studied here or by any of the Group II strains against the Group I *C. botulinum* strains, contrary to previous reports suggesting potential bactericidal activity by strain Alaska E [39]. Additionally, *C. botulinum* Group I and Group II strains dramatically affect each other’s toxin production if grown together, yet overall toxin production of a strain cocktail seems to remain high, similar to a previous report [68]. Future studies are needed to determine the environmental factors and molecular mechanisms underlying toxin production and growth competition between *C. botulinum* strains.

## Figures and Tables

**Table 1:** Strains used in this study.

Group	Strain	Toxin Subtype	Source	Connection to Foodborne Botulism Outbreak	Citation
Group I	56A	A	Canned Olives	Yes	[42]
	62A	A	Cow Liver Infarct	Unknown	[43,44]
	69A	A	Canned Spinach	Yes	[43,45]
	90A	A	Olives	Yes	[42]
	113B	B	Soil (Blue Ridge, KY, USA)	No	[42]
	213B	B	Canned Shallots	Yes	[44]
	Okra B	B	Home-Canned Okra Beans	Yes	[46]
Group II	Eklund 17B	B	Marine Sediments (Pacific Northwest, USA)	No	[43,47]
	Alaska E	E	Salmon Eggs	Yes	[47,48]
	Beluga E	E	Fermented Whale Flipper	Yes	[47]

**Table 2:** Inhibition between strains based on the well diffusion assay. Average radius of inhibition zone (n = 3): +: small (<2 mm), ++: medium (2–5 mm), and +++: large (>5 mm).

Test Strain										
Indicator Strain	56A	62A	69A	90A	113B	213B	Okra B	Eklund 17B	Alaska E	Beluga E
56A				+	+	+++				
62A				+	+++	+++				
69A	++	++		++		++				
90A					++	++				
113B	+	+		+		+++				
213B	++	+	+	+						
Okra B	+	+		+		+++				
Eklund 17B										
Alaska E						+				
Beluga E										

**Table 3:** BoNT activity of cultures grown at 30 °C for 4 days, determined by mouse bioassay.

	10-Strain Cocktail	9-Strain Cocktail	Group I Cocktail	Group II Cocktail	62A
**mLD_50_ (− trypsin)**	4.3 × 10^4^	4.3 × 10^4^	1.1 × 10^6^	<8 × 10^3 1^	1.1 × 10^6^
**mLD_50_ (+ trypsin)**	3.8 × 10^5^	1.1 × 10^6^	ND ^2^	1.2 × 10^5^	ND

^1^ No deaths by day 4 with the lowest dilution. ^2^ ND: not determined.

## Data Availability

Strain sequence data are available at NCBI/Genbank.

## Supplementary Materials

The following supporting information can be downloaded at: https://www.mdpi.com/article/10.3390/microorganisms10101895/s1, Figure S1: Well diffusion assay showing inhibitory activity between cocktail strains; Figure S2: Culture-streak assay showing inhibitory activity between cocktail strains; Figure S3: Western blot analyses of triplicate cocktails; Figure S4: Initial growth rate and final OD600 levels of individual strains and cocktails; Table S1: Accession numbers of strains used in this study; Table S2: Individual strain and cocktail pH.
