# Supplementary material for: Genomic Diversity, Competition, and Toxin Production by Group I and II Clostridium botulinum Strains Used in Food Challenge Studies
Source: Microorganisms. 2022 Sep 23;10(10):1895. doi: 10.3390/microorganisms10101895 (PMC9611418; doi:10.3390/microorganisms10101895)
Supplement: Supplementary file 1 [file microorganisms-10-01895-s001.zip › Supplementary Materials.pdf]

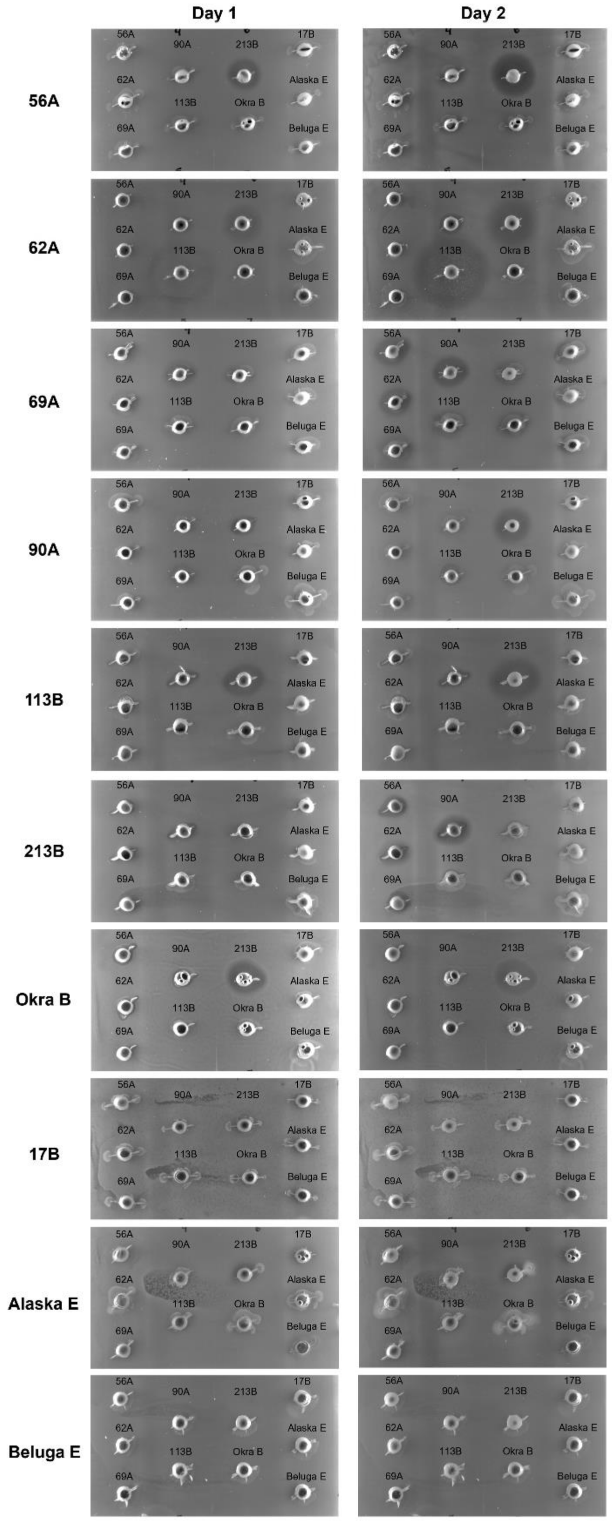

Figure S1. Well diffusion assay showing inhibitory activity between cocktail strains. Well diffusion assay through 2 days of incubation at 30°C demonstrating inhibition between strains. Zone of inhibition is classified based on the average radius of clearing in the lawn (n=3): small (<2mm), medium (2-5mm), large (>5mm). Strain Eklund 17B is abbreviated to 17B by the labeled wells.

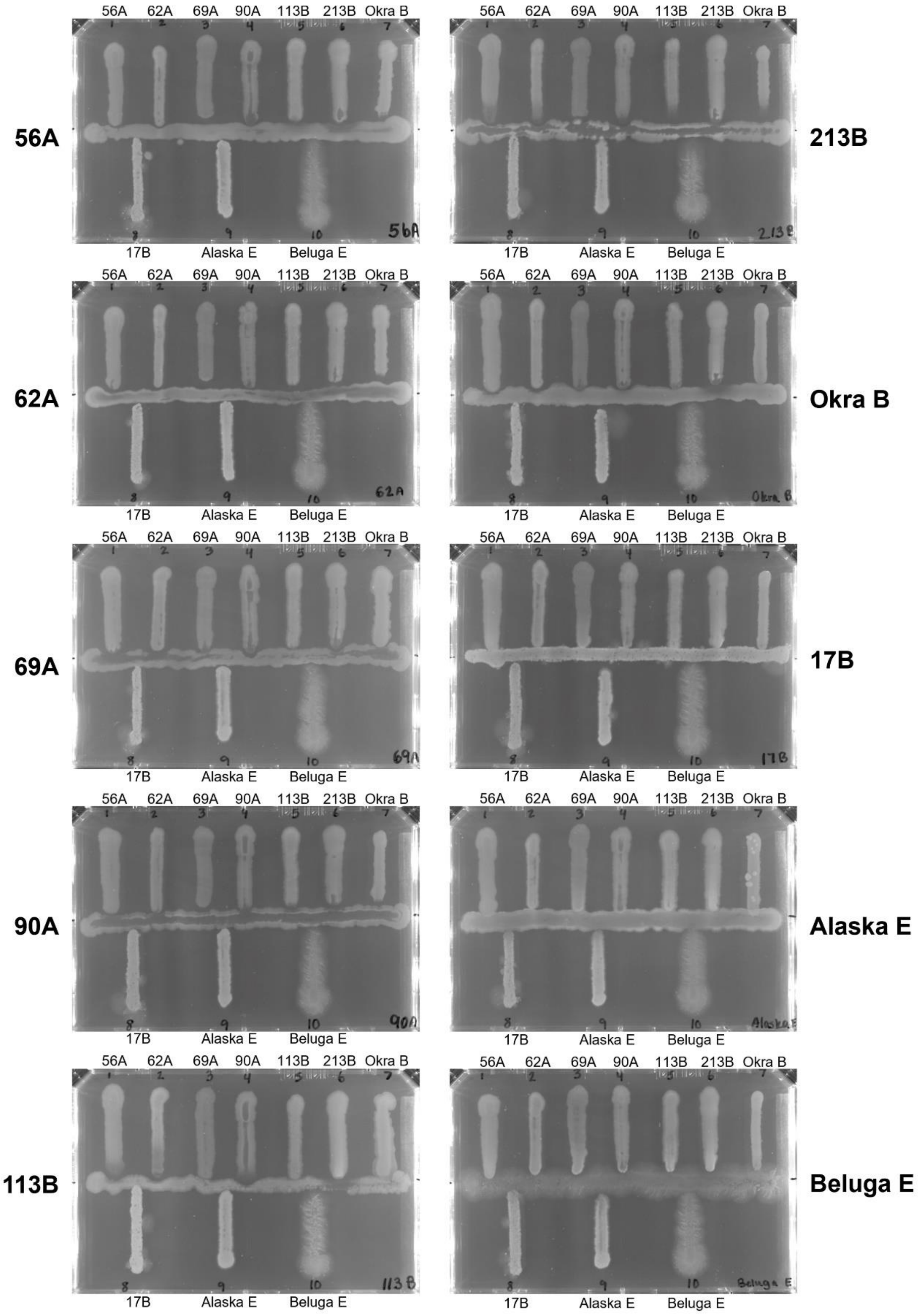

Figure S2. Culture-streak assay showing inhibitory activity between cocktail strains. Culture-streak assay for inhibition at 2 days of incubation at 30°C. Strain Eklund 17B is abbreviated to 17B by the labeled cultures.

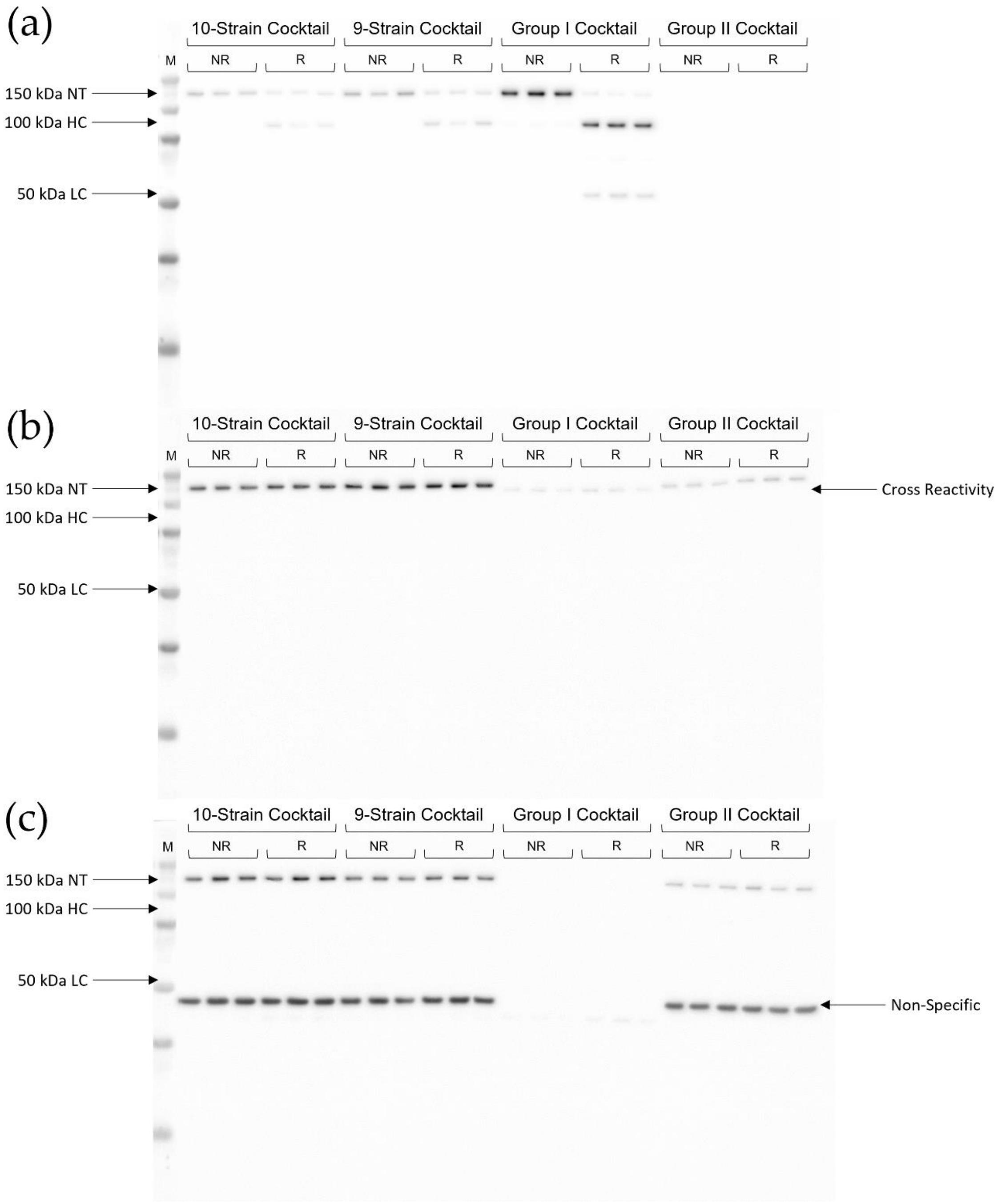

Figure S3. Western blot analyses of triplicate cocktails. Western blot analysis of non-reduced (NR) and reduced (R) whole culture cocktail samples alongside a molecular weight marker (M) (PageRuler Plus) grown for 4 days at 30°C in TPM. Toxin type produced were detected with **(a)** BoNT/A1-specific antibody, **(b)** BoNT/B1-specific antibody, and **(c)** BoNT/E3-specific antibody. A non-specific band can be seen on the membrane probed with antibody specific to BoNT/E **(c)**, and cross reactivity appears to have occurred in the Group I cocktail samples on the membrane probed with antibody specific to BoNT/B **(b)** slightly below where the expected 150 kDa band would be.

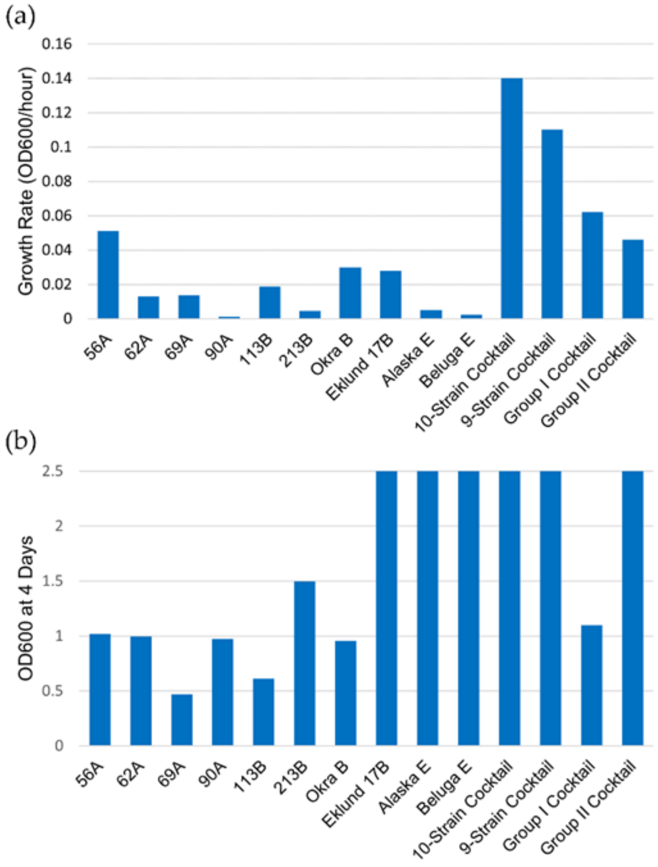

Figure S4. Initial growth rate and final OD600 levels of individual strains and cocktails. **(a)** Comparison of the initial growth rate of each individual strain and the average initial growth rate of each cocktail for the first 3.5 hours of incubation, calculated on excel using the OD600 values over the first 3.5 hours to determine the slope of the growth rate using linear regression. **(b)** Final OD600 measurement taken of each individual strain and the average of each cocktail mixture taken at 4 days of incubation. Differences in OD600 above 2.5 was not determined.

Table S2. Culture pH of individual strains and average pH of cocktail mixtures at 4 days of growth in TPM.

Strain	pH at 4 Days
56A	6.29
62A	6.31
69A	6.20
90A	6.34
113B	6.20
213B	6.24
Okra B	6.20
Eklund 17B	5.12
Alaska E	5.08

Beluga E	5.12
10-Strain Cocktail	5.86
9-Strain Cocktail	5.91
Group I Cocktail	6.13
Group II Cocktail	5.14
